# Supplementary material for: Radiogenomics in Renal Cancer Management—Current Evidence and Future Prospects
Source: Int J Mol Sci. 2023 Feb 27;24(5):4615. doi: 10.3390/ijms24054615 (PMC10003020; doi:10.3390/ijms24054615)
Supplement: Supplementary file 1 [file ijms-24-04615-s001.zip › Table S1.pdf]

Table S1. Radiomics studies on renal cell carcinoma management

Author/year	Study design/no patients	Primary outcome	Imaging modality and method	Results
Differentiation of benign from malignant kidney lesions				
Coy et al./2017 [243]	Retrospective/200	Discriminating benign lesions from cancerous lesions	- CT<br>- Semi-automated method using in-house-developed software (U.S. FDA 510K).	Characterization of ccRCC, chRCC, papRCC, ONC and AML had AUC of 0.850, 0.959, 0.792 and 0.825
Yu et al./2017 [22]	Retrospective/119	Discriminating benign lesions from cancerous lesions	- CT<br>- Semi-automated method with manual segmentation. MATLAB software to perform texture analysis. SVM for classifying different tumor types.	Histogram features skewness and kurtosis had the best discriminatory results (AUC 0.91 and 0.93, respectively). ML AUC 0.91-0.92.
Zhou et al./2019 [23]	Retrospective/192	Discriminating benign lesions from cancerous lesions	- CT<br>- Semi-automated method with manual segmentation. Preprocessing with Inception V3 software, pretrained on ImageNet and CNN models.	Model trained on slice dataset reported the worst result, with an ACC of 0.69. ROI dataset reported ACC of 0.97 while RBR had an ACC of 0.93.
Erdim et al./2020 [24]	Retrospective/79	Discriminating benign lesions from cancerous lesions	- CT<br>- Semi-automated method with manual segmentation. Feature extraction via MaZda software.	RF algorithm has been identified as having good prognostic potential with ACC of 0.905 and AUC of 0.905.
Uhlig et al./2020 [25]	Retrospective/94	Discriminating benign lesions from cancerous lesions	- CT<br>- Semi-automated method with manual segmentation. Feature selection with recursive feature elimination to build ML algorithms (RF) modeled to predict malignancy of specific renal mass.	AUC of RF was 0.83, better than expert radiologists (AUC 0.68).
Sun et	Retrospective/290	Discriminating	- CT	SVM model achieved a

al./2020 [26]		benign lesions from cancerous lesions	- Semi-automated method with semi-automated segmentation via Python v3.6.1 software. Radiomics was performed on VOI.	SENS ranging from 73% to 90% and a SPEC ranging from 89% to 91.7% in distinguish malignant from benign lesions.
Yap et al./2021 [244]	Retrospective/735	Discriminating benign lesions from cancerous lesions	- CT<br>- Semi-automated method with manual segmentation. 3D models used decision tree analysis (RF and REAL Adaboost)	Median AUCs 0.68-0.75 achieved by combined models.
Nassiri et al./2021 [28]	Prospective/684	Discriminating benign lesions from cancerous lesions	- CT<br>- Semi-automated method with manual segmentation. VOI and decision tree analysis model (RF and REAL Adaboost) has been used.	Prognostic model achieved an AUC of 0.84.
Said et al./2020 [29]	Retrospective/125	Discriminating benign lesions from cancerous lesions	- MRI<br>- Semi-automated method with manual segmentation. OsiriX software. Radiomics analysis was performed by MRI physicist utilizing MATLAB.	ML models (RF) with best results obtained an AUC of 0.73 in differentiate benign versus malignant lesions.
Xi et al./2020 [30]	Retrospective/1162	Discriminating benign lesions from cancerous lesions	- MRI<br>- Semi-automated method with manual segmentation.	Ensemble DL model reported the highest test ACC, SENS and SPEC, also when compared with the radiomics model.
Massa'a et al./2022 [31]	Retrospective/160	Discriminating benign lesions from cancerous lesions	- MRI<br>- Semi-automated method with semi-automated segmentation. HealthMyne software.	Best algorithm (SVM) had ACC of 0.80 and an AUC of 0.79.
Xu et al./2022 [32]	Retrospective/217	Discriminating benign lesions	- MRI<br>- Semi-automated	Best performance of DL model (combination of

		from cancerous lesions	method with manual segmentation. ROIs were manually outlined. DL used ResNet-18 architecture and radiomics models used RF	T2WI and DWI) AUC 0.925
Differentiation of angiomyolipoma from renal cell carcinoma				
Feng et al./2018 [33]	Retrospective/58	Differentiation of AML from RCC	- CT<br>- Semi-automated method with semi-automated segmentation via CT Kinetics software. Features were selected and classified via the SVM.	Optimal feature subset (using SVM and recursive feature elimination method), achieving an AUC of 0.955, SENS of 0.878 and SPEC of 0.1.
Cui et al./2019 [34]	Retrospective/169	Differentiation of AML from RCC	- CT<br>- Semi-automated method with semi-automated segmentation via ITK-SNAP v3.6.0 software. ROI was delineated manually. Texture analysis was performed via Python package PyRadiomics.	ML classifier (SVM and SMOTE) had best performance in differentiate AML from RCC types and ccRCC. AUC = 0.96 and 0.97, respectively.
Yang et al./2019 [35]	Retrospective/60	Differentiation of AML from RCC	- CT<br>- Semi-automated method with manual segmentation. Feature extraction was performed via Spyder 3.2.8 software.	Radiomics model (sRBFNN) yielded very good AUC (0.917), with an ACC, SENS and SPEC of, respectively, 0.90, 0.66 and 0.1.
Ma et al./2020 [36]	Retrospective/84	Differentiation of AML from RCC	- CT<br>- Semi-automated method with manual segmentation. ROI was manually delineated and depicted via the software ITK-SNAP v3.4.0.	Radiomics yielded an AUC of 0.988.
Nie et al./2020 [37]	Retrospective/99	Differentiation of AML from RCC	- CT<br>- Semi-automated method with manual	Radiomics training signature (AUC of 0.879) Validation signature (AUC

			segmentation. ROI was manually delineated and depicted via the software ITK-SNAP v3.8.	of 0.846).
Yang et al./2020 [38]	Retrospective/163	Differentiation of AML from RCC	- CT<br>- Semi-automated method with manual segmentation. ROI was manually delineated and depicted via the software ITK-SNAP. Features were extracted using Python package Pyradiomics.	SVM + t score and SVM + relief, had the best performance (AUC of 0.90).
Ma et al./2021 [39]	Retrospective/230	Differentiation of AML from RCC	- CT<br>- Semi-automated method with manual segmentation. VOI was depicted in ITK-SNAP software v3.4.0. Features were extracted via A.K. software, automatically.	Nephrographic and corticomedullary phase's radiomics experienced best (AUC of 0.726 versus 0.694 in the training set and 0.767 vs 0.754 in the validation set).
Ma et al./2021 [40]	Retrospective/139	Differentiation of AML from RCC	- CT<br>- Semi-automated method with manual segmentation. ROI was manually delineated and depicted via the software ITK-SNAP v3.4.0. 396 features were extracted using AK software.	Radiomics model reported an AUC of 0.975 in the training set and 0.923 in the validation set.
Han et al./2022 [41]	Retrospective/198	Differentiation of AML from RCC	- CT<br>- Semi-automated method with manual segmentation. VOI was delineated manually.	corticomedullary and nephrographic phases achieved an adequate performance after using multilayer perceptron classifier (AUC of 0.85, SENS of 0.76 and SPEC of 0.78 for the

				corticomedullary phase and an AUC of 0.83, SENS of 0.79 and SPEC of 0.78 for the nephrographic phase)
Kim et al./2022 [42]	Retrospective/84	Differentiation of AML from RCC	- CT<br>- Semi-automated method with semi-automated segmentation. Radiomics features were extracted using syngo.via Frontier software.	Radiomics model reached an AUC of 0.89 resembling with that of radiologists (AUC of 0.78).
Razik et al./2020 [43]	Retrospective/42	Differentiation of AML from RCC	- MRI<br>- Semi-automated method with manual segmentation. ROI was delineated manually. Features were extracted via TexRAD software.	Several texture parameters reported AUC > 0.8. The best performing parameter was mean of positive pixels, with an AUC of 0.891 on DWI.
Jian et al./2022 [44]	Retrospective/69	Differentiation of AML from RCC	- MRI<br>- Semi-automated method with manual segmentation via ITK-SNAP software v3.6.0. ROI was manually delineated. Features were extracted via AK software.	Radiomics model (AUC of 0.883) while Intravoxel incoherent motion-radiomics model (AUC of 0.874). Combined model (AUC of 0.919).
Matsumoto et al./2022 [45]	Retrospective/113	Differentiation of AML from RCC	- MRI<br>- Semi-automated method with manual segmentation. Radiomics features were extracted via LIFEx software v4.00. VOI was manually delineated.	Radiomics testing cohort (AUC of 0.90)<br>Validation cohort (AUC of 0.87)
Differentiation of oncocytoma from renal cell carcinoma				
Baghdadi et al./2020 [46]	Retrospective/192	differentiation of ccRCC from ONC	- CT<br>- Semi-automated method comprising 2 phases: (I) using DL network and manual tumor delineation;	SD Dice similarity score (0.66) for CNN model. PEER had ACC of 95% in tumor type classification (100% SENS and 89% SPEC) compared with the

			and (II) automated extraction of imaging features.	final pathology results.
Chen et al./2017 [27]	Retrospective/94	differentiation of ccRCC from ONC	- CT<br>- CT whole lesions and region of interest evaluated.	WL enhancement had poor results to differentiate between ccRCC and ONC (AUC of 0.78 and 0.72, respectively), Combination model (AUC of 0.86)
Coy et al./2019 [21]	Retrospective/179	differentiation of ccRCC from ONC	- CT<br>- CT imaging features with neural network model.	The best performance was obtained in the excretory phase (ACC of 74.4%, SENS =of 85.8% and PPV of 80.1%).
Deng et al./2020 [47]	Retrospective/501	Discriminating RCC from noncancerous renal lesions	- CT<br>- A ROI was drawn on venous phase axial CT. Different texture analysis parameters were compared between cohorts.	Differences in entropy were helpful in differentiation chrRCC from ONC.
Li et al./2020 [48]	Retrospective/61	Differentiation of chrRCC and ONC	- CT<br>- LASSO regression algorithm was used to analyze the CT image features. ROC curve and accuracy evaluation criteria.	1029 features extracted. Diagnostic performance (AUC >0.85); SVM classifier had the best performance (AUC 0.96; SENS 0.99; SPEC 0.80; ACC 0.94).
Raman et al./2014 [49]	Retrospective/99	Differentiation of ccRCC, papRCC, ONC and renal cysts	- CT<br>- ROIs were drawn manually. A predictive model using quantitative parameters was constructed and externally validated.	The RF model revealed 87% of ONC (SENS 89% and SPEC 99%). No AUC reported.
Sasaguri et al./2015 [50]	Retrospective/166	Differentiation of ONC versus RCC (papRCC and ccRCC and other subtypes)	- CT<br>- CT tumor attenuation values and texture parameters used in-house (Matlab (MathWorks) software. logistic regression model used	AUC 0.91 for differentiating ccRCC and other subtype RCCs from papillary RCCs.

			for differentiating types of renal lesions.	
Varghese et al./2018 [51]	Retrospective/174	Differentiation of malignant and benign renal masses (various subtypes)	- CT<br>- WL were manually segmented and co-registered from CECT scans.	Texture model had AUC of 0.87 ($p < 0.05$) for discriminating benign from cancerous kidney lesions.
Varghese et al./2018 [52]	Retrospective/156	Differentiation of malignant and benign renal masses (various subtypes)	- CT<br>- Manually segmentation of WL CT images 1. benign vs cancerous kidney lesions, 2. ONC vs ccRCC, and 3. ONC vs AML.	ROC analysis (AUC curve > 0.7, $p < 0.05$) between three groups.
Yu et al./2017 [22]	Retrospective/119	Differentiation of ccRCC, papRCC, chrRCC and ONC	- CT<br>- Manual segmentation of tumors. SVM method used for classification.	ML applied to texture analysis to differentiate ONC from other tumors (AUC of 0.86).
Hoang et al./2018 [59]	Retrospective/41	Differentiation of benign and cancerous kidney lesions (ONC vs ccRCC and papRCC) and RCC subtypes (ccRCC vs papRCC)	- MRI<br>- Texture features from WL MRI slides.	ONCs were distinguished from ccRCCs (SENS 67.3%, SPEC 88.9%, and ACC 79.3%), and from papRCC and ccRCCs (SENS 64.7%, SPEC 85.9%, and ACC 77.9%). No AUC reported.
Paschall et al./2018 [53]	Retrospective/55	Differentiation of ccRCC versus papRCC and ONC	- MRI<br>- WL measurements were performed. ROC curve analysis with optimal cutoff points was used to test the ability to the different groups.	WL ADC values were very different between papRCC and oncocytoma. Best AUC = 95.8 for ONC vs papRCC; SENS/SPEC 88.5% and 93.1% for ONC vs papRCC.
Differentiation of different subtypes of renal cell carcinoma				
Kocak et al./2018 [54]	Retrospective/68	- Differentiation of ccRCC, papRCC, and chRCC	- CT<br>- Feature selection was done by 3 radiologists. Feature selection and model optimization has been performed.	For differentiating non-ccRCCs from ccRCCs, the best performance was the ANN classifier (ACC of 84.6%). The performance was poor for

			Using artificial neural network and SVM as classifiers and a combination of 3 additional algorithms aimed to improve generalizability.	distinguishing ccRCC versus papRCC versus chRCC. Best the SVM classifier with bagging algorithm (ACC of 69.2%).
Han et al./2019 [55]	Retrospective/169	Differentiation of ccRCC, papRCC, and chRCC	- CT<br>- Rectangular ROI was marked and cropped. A DL neural network has been used to identify subtypes of RCC.	Deep DL method with the contouring given by radiologists for RCC subtype classification achieved an ACC 0.85, SENS 0.64-0.98, SPEC 0.83-0.93, and AUC 0.9
Li et al./2019 [56]	Retrospective/170 (training cohort) and 85 (validation cohort)	Differentiation of ccRCC from non-ccRCC	- CT<br>- 2 radiomics models were built. The radiogenomics association between selected features and VHL mutation has been analyzed. All models were independently validated.	The model from obtained from corticomedullary images from CT had AUC of 0.95 (ACC of 92.9%).
Raman et al./2014 [49]	Retrospective/99	Differentiation of ccRCC, papRCC, ONC and renal cysts	- CT<br>- ROIs were delineated in different phases of CECT images. Heterogeneity has been further assessed. A predictive model using quantitative parameters was constructed and externally validated.	Various renal masses (ONC, ccRCC, cysts, and papRCC) were accurately classified. The RF algorithm better categorized ccRCCs in 91% of images (SENS 91% and SPEC 97%), and papRCCs in 100% of cases (SENS 100% and SPEC 98%).
Leng et al./2017 [57]	Retrospective/139	Differentiation of ccRCC and papRCC and AML	- CT<br>- A largest possible ROI was manually drawn and SD, entropy, and uniformity were analyzed. Heterogeneity indices were further assessed with a denoising	Heterogeneity indices differentiated ccRCC from papRCC. Best AUC (0.91) for the subjective score.

			algorithm.	
Yan et al./2015 [58]	Retrospective/50	Differentiation of ccRCC and papRCC and AML	- CT<br>- Native and CECT images were analyzed and classified with texture analysis software (MaZda). Tumor attenuation values and enhancement degree was determined by a ROI.	For the discrimination between ccRCC and papRCC, excellent classification results were obtained with nonlinear discriminant analysis; on comparison of the 3 scanning phases, better lesion classification was observed with corticomedullary and nephrographic phase's images.
Hoang et al./2018 [59]	Retrospective/41	Differentiation of benign and cancerous kidney lesions (ONC vs ccRCC and papRCC) and different RCC subtypes (ccRCC vs papRCC)	- MRI<br>- The features obtained from native a contrast MRI images have been analyzed. Lasso regression used for false rate results.	papRCC was distinguished from ccRCC with an ACC of 77.9% (SENS 65.5% and SPEC 88.0%).
Li et al./2018 [60]	Retrospective/92	differentiation of ccRCC, papRCC, chrRCC, AML and ONC	- MRI<br>- ADC maps were constructed from FOV DWI images to identify the histogram parameters.	ADC histogram parameters differentiated eight of 10 pairs of renal tumors.
Paschall et al./2018 [53]	Retrospective/55	Differentiation of ccRCC versus papRCC and ONC	- MRI<br>- WL measurements were performed, and ADC map constructed from WL histogram.	WL ADC features could discriminate papRCC from ONC. Best percentile ROC analysis demonstrated AUC of 95.2 (sensitivity of 84.5% and specificity of 93.1%).
Fuhrman grade prediction				
Bektas et al./2018 [61]	Retrospective/54	High-grade (Fuhrman 3-4) tumor prediction	- CT<br>- ML-based SMV, naïve Bayes, k-nearest neighbors, and RF classifiers for quantitative 2D TA	SMV model predicted high-grade pathology with 85.1% overall ACC, 91.3% SENS, 80.6% SPEC, and AUC of 0.860.
Deng et	Retrospective/114	Detection of	- CT	Texture-score AUC 0.843.

al./2018 [47]	training cohort and 92 validation cohort	high-grade (Fuhrman 3-4) tumor	- ML-based LASSO to select features and build a texture-score	Non-TA features (round mass, diameter, artery tumor, relative tumor enhancement value) were compared to TA features and round mass was similar (AUC 0.723). Prediction model including both texture and non-texture features did not outperform that including solely TA features in both cohorts.
Shu et al./2018 [63]	Retrospective/260	High-grade (Fuhrman 3-4) tumor prediction	- CT<br>- LASSO for feature selection. Models built by LR	3 models were created using features from CMP, NP, or CMP + NP. CMP model's ACC was 71.9%, AUC 0.766, SENS 0.602, and SPEC 0.838; NP model's ACC was 73.8%, AUC 0.818, sSENS 0.693, and SPEC 0.838; and CMP + NP model's ACC was 77.7%, AUC 0.822, SENS 0.677, and SPEC 0.839. The CMP + NP model's AUC was significantly higher than that of CMP alone and all other AUCs were similar between them.
Gill et al./2019 [64]	Retrospective/84	Differentiating juxtatumoral perinephric fat of high-grade (ISUP 3-4) versus low-grade (ISUP 1-2)	- CT<br>- Radiomics panel of tissue characterization	All TA methods but gray-level difference matrix showed differences and increased heterogeneity index in high-grade juxtatumoral perinephric fat.The measure of correlation coefficient form GLCOM had the best accuracy (AUC 0.746).
Goyal et al./2019 [65]	Retrospective/29	High-grade (Fuhrman 3-4) tumor detection	- MRI<br>- ML-based TexRAD arranging according to size in SSF	The best performance was found in Entropy (at SSF 6 on diffusion-weighted image) AUC: 0823 (0.618–1.0), mean (at SSF 3 on CMP) AUC:

				0.889 (0.655–1.9), and mean of positive pixels (at SSF 5 on NP) AUC: 0.870 (0.712–1.0)
He et al./2019 [66]	Retrospective/227	Prediction accuracy of high-grade (ISUP 3–4) tumors by 5 TA-based models	- CT<br>- ML-based on ANN fed with radiomics signatures prediction models	5 models based on features with the best performance had a predictive mean value of 92.46%. The top-ranked model was a combination of minimum mean squared error of conventional image features and CMP phase (94.06%)
Kocak et al./2019 [67]	Retrospective/91	High-grade (Fuhrman 3-4) tumor detection	- CT<br>- ANN and binary LR with and without SMOTE	The ANN algorithm (based on 5 TA features) outperformed that of logistic regression (based on 6 features). ANN algorithm detected 81.5% of high-grade tumors accurately (AUC 0.714).
Lin et al./2019 [68]	Retrospective/232	High-grade (Fuhrman 3-4) tumor detection	- CT<br>- ML-based CatBoost	The ML model based on 3-phase CT images detected high-grade tumors with an AUC 0.87, outperforming those based on single-phase images.
Shu et al./2019 [63]	Retrospective/271	High-grade (ISUP 3-4) tumor prediction	- CT<br>- LASSO for feature selection. The k-nearest neighbor, LR, MLP, RF, and SVM as ML-based classifiers	The best model was achieved by the combined classifier (CMP + NP features) with 91.7-93.5% ACC and an AUC of 0.96-0.98 in the validation cohort compared to the training cohort with 86.5-90.8% ACC and an AUC of 0.950.97.
Sun et al./2019 [69]	Retrospective/227	High-grade (ISUP 3-4) tumor prediction	- CT<br>- ML-based SMV. Variant selection and LASSO for feature selection	A model combining features of both phases (CMP and NP) with SMV classifier achieved best performance in the training and validation datasets, with an AUC of 0.88 (0.77–0.95; SENS 0.85 and SPEC 0.89) and

				0.91 (0.65–0.99, SENS 0.83 and SPEC 0.89), respectively.
Cui et al./2020 [70]	Retrospective/460	Comparison between CECT- and MR-based high-grade (ISUP 3-4) prediction; high-grade accuracy prediction and external validation	- CT and MRI<br>- ML-based CatBoost	MRI ML-TA accuracy did not outperform that of CT either in the internal (79% vs 73%) or in the external (69% vs 74%) cohorts' datasets. CECT and MRI multiphase TA improved accuracy prediction 2-10% compared to single-phase. Similar results between cohort datasets were reported.
Assessment of treatment response in metastatic RCC				
Antunes et al./2016 [71]	Prospective/2	Modifications of radiomics features after Sunitinib therapy	- FLT-PET/MRI<br>- Image related features before and after treatment using [18 F] FLT-PET, T2w, and DWI protocols, with DWI reporting an ADC map.	The best radiomics yielded a modification of 63% within the RCC region and 17% in a distinct normal region.
Bharwani et al./2014 [72]	Retrospective/20	Changes in histogram parameters and correlation with OS (changes prior and after treatment with Sunitinib)	- MRI<br>- ADC maps and histograms have been assessed. Mean ADC and proportion of the tumor with ADC values <25th percentile of ADC histogram were recorded. ROI were manually delineated. Changes prior and after therapy in surviving patients have been compared for OS.	Outcomes did not correlate to features. High baseline AUC low and greater median AUC low have been associated with poor OS (p=0.038). OS had no correlation with MRI features.
Boos et al./2017 [73]	Retrospective/19	Changes in CT intensity distribution curves in measurable soft tissue lesion with	- CT<br>- Histograms delineated form ROI. Shift was used to classify response of lesions to therapy and any modifications on	Changes in histograms appeared in in 58% of lesions, and a significant difference between mean and median lesion attenuation (p < 0.001). There has been an

		sunitinib and sorafenib	scans, using the Choi, MASS and RECIST criteria.	increased in changes of the accurate classification of tumors when Choi and Mass criteria were evaluated (63% to 68% and 74% to 79%).
Goh et al./2011 [74]	Retrospective/39	Changes in histogram parameters (entropy and uniformity) and correlation of texture parameters with PFS in pts treated with sunitinib, cedirinib, pazopanib, and regorafenib	- CT<br>- A CAD software algorithm appreciated the changes in entropy and uniformity of metastasis. RECIST, Choi and modified Choi criteria evaluated the response. The correlations of texture parameters and standard criteria with PFS have been assessed.	Tumor entropy decreased and uniformity increased following TKI therapy. Kaplan-Meier curves of patients without disease progression reported better outcomes compared to standard response assessment (p=0 .008 vs 0.267, p=0 .053, and p= 0.042 for RECIST, Choi, and modified Choi criteria, respectively). Texture uniformity was an independent predictor of time to progression (p= 0.005).
Haider et al./2017 [75]	Retrospective/40	Correlation of texture parameters with OS and PFS in ccRCC treated with Sunitinib	- CT<br>- Measurable lesions on CECT before and 2 months after therapy. TexRAD software (TexRAD Ltd, Cambridge, UK) has been employed to analyze textures. Cox regression model assessed changes in texture features and PFS/OS.	Size normalized SD (nSD) alone is good predictor of OS (p = 0.01). Entropy modifications are a good predictor for OS (p= 0.02 and p= 0.04) and nSD can prognoses PFS (p= 0.01 and p= 0.003).
Mains et al./2018 [76]	Retrospective/69	Association between OS and PFS with functional CT parameters (various treatments, not specified)	- CT<br>- Scans performed at prior and after therapy (after 5 and 10 weeks). BVdeconv, BFdeconv, SPVdeconv, blood flow and standardized perfusion values (BFmax and SPVmax), were evaluated using	The strongest association was found for BVdeconv, BVpatlak and BFdeconv prior and after therapy ($p < 0.05$). PS seemed to have opposite associations dependent on treatment. Inter-observer correlations were excellent ($r \geq 0.9$, $p < 0.001$) with good agreement for BFdeconv,

			the Patlak model (BVpatlak and PS).	BFmax, SPVdeconv and SPVmax.
Khene et al./2021 [77]	Retrospective/48	Predict response to Nivolumab tretament	- CT<br>- K-nearest neighbor, RF, logistic regression, and SVM algorithms have been used. Classification of patients: complete or partial response or stable disease and non-responders.	95% of patients received nivolumab. 60.4% of patients were nivolumab responders. The ACC (0.71 till 0.91) and the AUC (0.67 till 0.92). RF reported the worse ACC, while logistic regression the highest.

RCC= renal cell carcinoma; ccRCC= clear cells RCC; chRCC= chromophobe RCC; papRCC= papillary RCC; AML= angiomyolipoma; CT= computed tomography; CNN= convolutional neural network; MRI= magnetic resonance imaging; 3D= three-dimensional; 2D= two-dimensional; WL= whole lesion; ROI= region of interest; SD= standard deviation; ROI= receiver operating characteristic; ROC= Receiver operator characteristics; AUC= area under the curve; ACC= accuracy; SENS= sensitivity; SPEC= specificity; PPV= positive predictive value; VOI= volume of interest; LASSO= least absolute shrinkage and selection operator; ONC= oncocytoma; SVM= support vector machine; ADC= apparent diffusion coefficient; DL= deep learning; ML= machine learning; CECT= contrast enhanced computed tomography; RBR= rectangular box region; RF= random forest; **SMOTE**= synthetic minority oversampling technique**;** sRBFNN= self-organized radial basis function network; PEER= peak early-phase enhancement ratio; DWI= diffusion-weighted imaging; FOV= field of view;
