# Supplementary material for: Radiogenomics in Renal Cancer Management—Current Evidence and Future Prospects
Source: Int J Mol Sci. 2023 Feb 27;24(5):4615. doi: 10.3390/ijms24054615 (PMC10003020; doi:10.3390/ijms24054615)
Supplement: Supplementary file 1 [file ijms-24-04615-s001.zip › Table S2.pdf]

Table S2. Main MI modalities for RCC management.

MI modality	Potential Application	Metabolic pathway and Main Features	Limitations
^{99m}Tc-sestamibi SPECT/CT	Detection of ONC and HOCT	- Accumulates in cells with high mitochondrial content and low MRP expression	- Low spatial resolution
^{124}I- and ^{89}Zr-girentuximab PET/TC	Detection of ccRCC, treatment response	- Targets CAIX (overexpressed in 95% ccRCC)<br>- Advantages over SPECT/CT (e.g., contrast and spatial resolution)	- Long interval between radiotracer injection and PET/CT scan (3-7 days)<br>- High cost
^{18}F-FDG PET/CT	Detection of recurrent and metastatic RCC, prognosis and treatment response assessment	- Glycolysis and pentose phosphate pathway<br>- Targets malignant cells with increased aerobic glycolysis<br>- Readily available	- Physiological renal excretion<br>- Some RCC exhibit a low ^{18}F-FDG uptake<br>- Non-specifically taken up by any malignant cells with cytosolic aerobic glycolysis
^{68}Ga-PSMA-11 PET/CT	Differentiation between malignant and benign lesions	- PSMA is overexpressed in tumor-associated neovascular endothelial cells of numerous solid tumors<br>- Readily available	- High accumulation in the kidney
^{11}C-acetate PET/CT	Differentiation between malignant and benign lesions	- involved in numerous metabolic processes, including lipid synthesis (significantly upregulated in RCC)	- RCC shows high heterogeneity for acetate uptake

MI= molecular imaging; RCC= renal cell carcinoma; SPECT= single photon emission computed tomography; PET= positron emission tomography; CT= computed tomography; PSMA= prostate-specific membrane antigen; ^{18}F-FDG= [^{18}F] fluoro-D-glucose; ONC= oncocytoma; HOCT= hybrid oncocytic/chromophobe tumor; ccRCC=clear cell RCC; MRP= multidrug resistance pump; CAIX= carbonic anhydrase IX.
