# Supplementary material for: Radiogenomics in Renal Cancer Management—Current Evidence and Future Prospects
Source: Int J Mol Sci. 2023 Feb 27;24(5):4615. doi: 10.3390/ijms24054615 (PMC10003020; doi:10.3390/ijms24054615)
Supplement: Supplementary file 1 [file ijms-24-04615-s001.zip › Table S3.pdf]

Table S3 Radiogenomics studies on clear cell renal cell carcinoma

**Radiomics features association with mutational status**	**Author/Year**	**Study design/no patients**	**Primary outcome**	**Results**
	Karlo et al./2014 [204]	Retrospective/233	Association of radiomics CT images and gene mutations	Correlation with good defined tumor edges (p=0.013), nodular tumor appearance (p=0.021), brut enhancement of vascularity (p=0.018) for VHL mutation and renal vein invasion p=0.022 for KDM5C and p=0.046 for BAP1. Mutations of SETD2, KDM5C and BAP1 were absent in multicystic lesions. Mutations of VHL and PBRM were associated with solid lesions.
	Shinagare et al./2015 [214]	Retrospective/103	Association of radiomics CT images and gene mutations	BAP1 mutation correlated with poor defined tumor edges (p = 0.02 and calcification (p=0.002). MUC4 mutation correlated with exophytic development (p = 0.002)
	Bowen et al./2018 [215]	Retrospective/177	Association of radiomics CT images and molecular m1 to m4 subtypes	m1 predicted good defined margins and m3 predicted less accurate defined margins, the probability of collector system and renal vein involvement.
	Marigliano et al./2019 [216]	Pilot retrospective/20	Association of radiomics CT images and miRNA expression	An association of entropy and miR-21-5p expression has been determined between ccRCC and benign tissue
	Cianflone et al./2022 [217]	Pilot retrospective/6	Association of radiomics CT images and gene expression	Four radiogenomics models have been identified to have a significant correlation between radiomics and transcriptomics.
Prediction of genes mutation status				
	Ghosh et al.2015 [205]	Retrospective/78	Association of radiomics CECT features to predict	Random forest model obtained AUC between 0.52 and 0.71 (best for nephrographic phase)

			BAP1 mutation status	for BAP1 mutation prediction.
	Kocak et al./2019 [206]	Retrospective/45	ML CT radiomics features to predict PRBM1 mutation status	Best ACC and AUC for random forest models (95.0%, AUC = 0.987)
	Kocak et al.2020 [207]	Retrospective/65	ML CT radiomics features to predict BAP1 mutation status	Model SPEC of 78.8% and PREC of 81% for presence and absence of BAP1 mutations, respectively
	Chen et al.2019 [208]	Retrospective/57	ML CT radiomics features to predict gene mutation status	The SENS, SPEC and PREC for prediction of BAP1 mutation (90.4%, 78.8%, and 81%). The SENS, SPEC and PREC for prediction of BAP1 absent mutation (78.8%, 90.4%, and 89.1%)
	Yin et al./2019 [210]	Retrospective/23	Multi-omics incorporating PET/MRI radiomics model to predict ccA or ccB molecular subtype	Correct classification rate (radiomics alone 86.96 vs. multi-omics 95.65%)
	Feng et al./2020 [209]	Retrospective/54	ML CT radiomics features to predict BAP 1 mutation status	The prediction of BAP1 mutation achieved an AUC = 0.77, with SENS of 0.72, SPEC of 0.87, and PREC of 0.65
Prediction of clinical outcomes				
	Jamshidi et al./2015 [225]	Retrospective/70	Radiogenomics risk score from CT image to predict gene expression and disease specific survival	Significant association of radiogenomic risk score with DSS (log-rank $p < 0.001$)
	Jamshidi et al./2016 [220]	Prospective/41	Radiogenomics risk score predictive of radiologic progression free survival in immunotherapy neoadjuvant setting	A low RRS vs high RRS leads to a higher rPFS and OS when compared with high RRS (25 months vs. 6 months, $p=0.005$) and (37 months vs. 25 months, $p=0.03$)

	Cen et al./2019 [224]	Retrospective/106	Association of CT radiomics features and RUNX3 methylation level and survival	Independent predictors of high RUNX3 methylation are good vs. poor defined tumor edges and present vs absent intratumoral vascularity, AUC=0.725. Low RUNX3 methylation is associated with longer median OS (p<0.001)
	Yu et al./2020 [219]	Retrospective/212	Association of CT radiomics features and DNA methylation subtyping and effects on overall survival	The M1 subtype is associated with poor median OS (p<0.001)
	Lee et al./2020 [211]	Pilot retrospective/58	Multiphase CT radiomics model to predict metastases and WTS gene expression	Radiogenomics approach achieved an AUC=0.955 for metastasis prediction
	Lin et al./2021 [212]	Retrospective/160	ML techniques to identify CT radiomics subtypes that have clinical involvement	C1 radiomics subtype with related mutation frequency revealed a lower OS (p=0.027) and PFI (p=0.002) than the C2 and C3 subtypes.
	Zhao et al./2021 [213]	Retrospective/547	ML radiomics features to predict distant metastasis and gene modules associated with radiomics features	AUC 0.95 for metastasis prediction
	Udayakumar et al./2021 [229]	Prospective/49	Spatial DCE-MRI imaging features for prediction of molecular subtypes	High PFS with TKI in tumors with high compared with low MRI vascularity (p<0.0001) and no difference in IO patients.
	Huang et al./2021 [218]	Retrospective/205	OS prediction through a radiogenomics model	Imaging-genomics prognostic factors achieved better AUCs vs imaging alone at 1, 3 and 5 years (AUC=0.814 vs. 0.837, 0.74 vs. 0.806, and 0.689 vs. 0.751)
	Zeng et al./2021 [221]	Retrospective/207	Association of CT radiomics features	The multiple omics model achieved best 5 years OS AUC

			and multiple omics and survival outcomes	(0.846)
	Gao et al./2022 [222]	Retrospective/520	Prognostic radiogenomics marker to predict gene expression and stratification of patients survival	Radiogenomics model (radiomics features and immune-related gene signature expression levels) had an AUC of 0.76 in the training group and 0.72 in the validation group
	Gao et al./2022 [223]	Retrospective/521	Prognostic radiogenomics marker and hypoxia related genes for prognosis prediction	Radiomics features and hypoxia genomics signature is associated with survival (AUC=0.91, both in training and validation group) and externally validated (p=0.0059)

ACC= accuracy; AUC= area under the curve; ccRCC= clear cell RCC; CT= computed tomography; CECT= contrast enhanced computed tomography; DCE-MRI= dynamic contrast-enhanced magnetic resonance imaging; DSS= disease specific survival; miRNA= micro ribonucleic acid; ML= machine learning; MRI= magnetic resonance imaging; OS= overall survival; PET/MRI= positron emission tomography/magnetic resonance imaging; PFS= progression free-survival; rPFS = radiological progression free survival; PREC= precision; RCC= renal cell carcinoma; RRS= radiogenomics risk score; SENS= sensitivity; SPEC= specificity; TKI= tyrosine kinase inhibitor; WTS= whole transcriptome sequencing;
